# Acute effects of coffee on QT interval in healthy subjects

**DOI:** 10.1186/1475-2891-10-15

**Published:** 2011-02-02

**Authors:** Silvio Buscemi, Alessandro Mattina, Maria Rosaria Tranchina, Salvatore Verga

**Affiliations:** 1 Department of Medicina Interna, Malattie Cardiovascolari e NefroUrologiche; Faculty of Medicine, University of Palermo, Italy

## Abstract

The coronary endothelial function is recognized to have an important role in the physiology of the diastolic ventricular relaxation, a phase of the heart cycle that influences the electrocardiographic QT interval. Endothelial function is investigated *in vivo *by flow mediated dilation (FMD) in the brachial artery and has proven to be a strong predictor of both coronary endothelial function and cardiovascular events. It has been reported that coffee acutely induces FMD changes. In particular, the brachial artery FMD seems to decrease after caffeinated coffee (CC) and to increase after decaffeinated coffee (DC) ingestion. Since the cardiovascular effects of coffee are still a debated matter, this study aimed at investigating with a randomized, double-blind crossover design, if the QT interval of adult healthy subjects (19 males and 21 females) changes in the hour following CC or DC ingestion. Both systolic and diastolic blood pressure were higher in the hour following the ingestion of CC; the heart rate significantly increased 30 minutes after CC ingestion. A significant increase of the QT duration was observed one hour after DC ingestion (398.9 ± 3.8 vs 405.3 ± 3.7 msec; P < 0.05), not after CC. The QT interval corrected for heart rate did not significantly change following CC or DC ingestion. In conclusion, despite CC and DC previously demonstrated to influence the FMD they do not seem to induce a significant unfavourable acute change of the left ventricular repolarization. Further investigations are required to elucidate the effects of coffee in subjects with cardiovascular diseases.

## Findings

Many controversies exist about the cardiovascular effects of coffee in man. We recently demonstrated [1] that endothelial function in healthy subjects, measured using flow-mediated dilation (FMD) of the brachial artery, is significantly lower in the hour following the ingestion of 25 ml of espresso coffee. On the contrary, there was a dose-dependent significant increase in FMD when one (25 ml) or two (50 ml) cups of decaffeinated coffee were ingested [1,2]. We attributed these differences to the opposite effects of caffeine and anti-oxidant substances contained in coffee mixtures. The coronary endothelial function is recognized to have an important role in the physiology of the diastolic ventricular relaxation, therefore, it cannot be excluded that the effects of coffee on the FMD in the brachial artery are of some relevance also for both the coronary bed and the diastolic function. The electrocardiographic QT interval explores the repolarization that occurs during the left ventricular diastolic phase; furthermore, the QT corrected for heart rate (QTc) has been correlated with both atherosclerotic disease and cardiovascular mortality. It has been reported that caffeine ingestion unfavourably affects the QT interval during sleeping in adult healthy subjects [3], however, another study did not evidence any significant influence of caffeine ingestion on the QT interval [4]. Since no study has directly investigated the effect of coffee on the QT interval, we compared the acute effects of caffeinated coffee (CC) vs decaffeinated coffee (DC) on the QT interval in adult healthy subjects.

The study design has been previously described elsewhere [1]. The study was approved by the ethics committee of the University Hospital Policlinico P. Giaccone of Palermo, Italy, and registered as an International Standardized Randomized Controlled Trial (ISRCTN85096812). An approved informed consent form was signed by each subject.. Briefly, 40 volunteers (19 males and 21 females) aged 21-49 years and with body mass index (BMI, (body weight (kg)/height (m)^2^) between 17.3-28.0 kg/m^2 ^were included in the study. Exclusion criteria were any dyslipidemia, hypertension, diabetes, cardiovascular or systemic disease, any medication treatment, smoking of any tobacco products, pregnancy or lactation in the past 6 months, habitual daily consumption of greater than two cups of coffee or weekly ingestion of more than one commercial caffeinated beverage and abstaining from chocolate or other flavonoid-containing beverages up to the preceding day. The study followed a randomized, crossover, double-blind design with each subject receiving two different study treatments, in random order, and repeated on separate days at 5- to 7-day intervals. The preparation of either CC and DC has already been described elsewhere [1]. Each packet of CC or DC contained a mixture of 65% Robusta (variety Canephora) and Arabica (A. Morettino s.p.a.; Palermo, Italy). The average caffeine content in 25 ml of CC and DC measured by chromatography-spectrophotometry (Chemical Laboratory, Camera di Commercio Industria Artigianato e Agricoltura, Trieste, Italy) was 130 mg and 5 mg, respectively. No addition of sugar or milk was permitted. Subjects had continuous electrocardiogram (ECG) and blood pressure (10 min intervals) recorded for the duration of each test. Three computed standard 12-lead ECG (Mortara Rangoni, Bologna, Italy) were performed at three minutes intervals before and one hour after coffee ingestion; both the QT and QTc (Bazzett's formula) intervals of each ECG were automatically measured using a dedicated software and the mean of the three ECGs was considered for the two measurement periods.

All data are presented as means ± standard error of the means. Basal pairwise comparisons between the two treatments (CC vs DC) were tested for statistical significance using the paired Student's t-test. An overall 3 × 2 ANOVA (analysis of variance) for repeated measures was performed to evaluate the composite effect of the two different (CC and DC) ingested coffees over time (three periods: baseline, and 30 and 60 min) on the parameters of interest. ANOVA for repeated measures was also carried out separately to detect significant changes in variables over time within the two sessions; Bonferroni's t-test was performed for individual differences between two time points (paired) when appropriate. A two-tailed P < 0.05 was considered significant. All analyses were performed using Systat (Windows version 11.0; San Jose, CA, USA).

**Figure 1:**
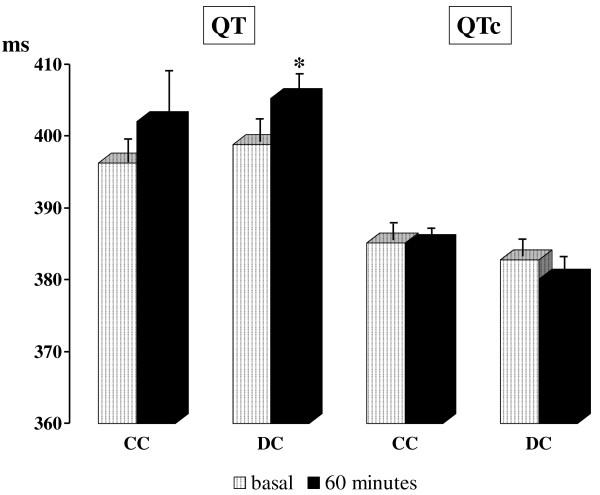
**QT and heart rate corrected QT (QTc) intervals before (white bars) and 60 min after (dark bars) ingestion of one cup of caffeinated (CC) or decaffeinated (DC) espresso coffee**. Data are expressed as mean ± SEM and analyzed by using paired t-test. * P < 0.05 for the comparison versus baseline.

Both systolic and diastolic blood pressure were higher in the hour following the ingestion of CC with a time × treatment effect (Table 1). This result confirms our previous study [1] with the exception of the time × treatment effect that previously was not observed probably due to the limited number of studied subjects in that circumstance (20 subjects vs 40 subjects in the present study). Also we confirm that the heart rate significantly increased 30 minutes after CC ingestion as in our previous study [1]. The Figure 1 reports the changes in QT and QTc intervals after CC or DC ingestion. A significant increase of the QT duration was observed one hour after DC ingestion (398.9 ± 3.8 vs 405.3 ± 3.7 msec; P < 0.05), not after CC. This result is of uncertain interpretation but it is probably influenced by changes in heart rate. In fact the QTc duration did not significantly change after either CC or DC ingestion. Therefore, despite the same mixtures of CC and DC previously demonstrated to have significant influences on FMD [1,2], they do not seem to have a significant unfavourable acute influence on left ventricular repolarization. This result is limited to healthy subjects and need to be confirmed in other more vulnerable cohorts as those characterized by the presence of cardiovascular risk factors or already established cardiovascular diseases. Further studies are needed to explore other possible cardiovascular effects of coffee. In particular, it would be interesting to analyse the QT interval dispersion and the echocardiographic measures of diastolic dysfunction. In fact, it has been recently reported [5] that caffeine is responsible of a reduced myocardial blood flow response to physical exercise in patients with coronary artery disease. The possibility also exists that caffeine may influence both left ventricular repolarization and diastolic function modifying the sympathetic/parasympathetic balance, however no hormonal or instrumental measures were considered in this study in order to investigate this hypothesis.

**Table 1:** Changes in vital signs following ingestion of caffeinated or decaffeinated espresso coffee1

	Coffee		**P-value**^**2**^	
	Caffeinated	Decaffeinated	Time	Time × Treatment
		
	N = 40	N = 40		
Systolic blood pressure (mmHg)				
basal	107 ± 2	107 ± 2		
30 min	112 ± 2^a^	106 ± 2	<0.05	<0.001
60 min	111 ± 2^a^	107 ± 2		
			
P-value^3^	<0.001	0.50		
Diastolic blood pressure (mmHg)				
basal	68 ± 1	67 ± 1		
30 min	72 ± 1^a^	66 ± 1	<0.001	<0.005
60 min	71 ± 1^b^	68 ± 1		
			
P-value^3^	<0.001	0.16		
Heart rate (beats/min)				
basal	67 ± 1	66 ± 1		
30 min	69 ± 1^c^	67 ± 1	<0.001	0.16
60 min	64 ± 1^d^	65 ± 1^e^		
			
P-value^3^	<0.001	0.09		

^1 ^All values are mean ± SEM. ^2 ^3 × 2 ANOVA for repeated measures.

^3 ^- P-value compares within group values between basal, 30 min, and 60 min for each variable within each group.

Paired t-test: ^a ^*P *< 0.001 vs. both decaffeinated coffee and basal value. ^b ^*P *< 0.001 vs. basal value and *P *< 0.05 vs. decaffeinated coffee. ^c ^*P *< 0.05 vs. basal value. ^d ^*P *< 0.005 vs. basal value and *P *< 0.001 vs 30 min value; ^e ^*P *< 0.05 vs 30 min value.

We conclude that despite CC acutely increases systolic and diastolic blood pressure as well as heart rate, both caffeinated and decaffeinated coffee do not acutely induce any significant change in the QTc interval duration in healthy adult subjects.

## List of abbreviations

BMI: body mass index; CC: caffeinated coffee; DC: decaffeinated coffee; ECG: electrocardiogram; FMD: flow-mediated dilation; QTc: QT interval corrected for heart rate;

## Competing interests

The authors declare that they have no competing interests.

## Authors' contributions

SB was the main author of the manuscript and contributed to the design of the study, preparation of protocols, statistical analyses, interpretation of data and preparation of the manuscript. AM carried out all the experiments, contributed to the interpretation of data and preparation of the manuscript. MRT carried out all the experiments, contributed to the preparation of the manuscript. SV contributed to interpretation of data and preparation of the manuscript. All authors read and approved the final manuscript.
